# The S_1_ to S_2_ and S_2_ to S_3_ state transitions in plant photosystem II: relevance to the functional and structural heterogeneity of the water oxidizing complex

**DOI:** 10.1007/s11120-024-01096-4

**Published:** 2024-04-25

**Authors:** Andrea Pavlou, Stenbjörn Styring, Fikret Mamedov

**Affiliations:** Molecular Biomimetics, Department of Chemistry-Ångström, Uppsala University, P.O. Box 523, 751 20 Uppsala, Sweden

**Keywords:** Photosystem II, Water oxidation, S state transitions, Low spin S_2_ state, High spin S_2_ state, Temperature dependence, Electron paramagnetic resonance

## Abstract

In Photosystem II, light-induced water splitting occurs via the S state cycle of the CaMn_4_O_5_-cluster. To understand the role of various possible conformations of the CaMn_4_O_5_-cluster in this process, the temperature dependence of the S_1_ → S_2_ and S_2_ → S_3_ state transitions, induced by saturating laser flashes, was studied in spinach photosystem II membrane preparations under different conditions. The S_1_ → S_2_ transition temperature dependence was shown to be much dependent on the type of the cryoprotectant and presence of 3.5% methanol, resulting in the variation of transition half-inhibition temperature by 50 K. No similar effect was observed for the S_2_ → S_3_ state transition, for which we also show that both the low spin g = 2.0 multiline and high spin g = 4.1 EPR configurations of the S_2_ state advance with similar efficiency to the S_3_ state, both showing a transition half-inhibition temperature of 240 K. This was further confirmed by following the appearance of the Split S_3_ EPR signal. The results are discussed in relevance to the functional and structural heterogeneity of the water oxidizing complex intermediates in photosystem II.

## Introduction

Photosynthesis is an important biological process which converts solar energy into chemical energy which then is widely used in the biosphere. Photosystem II (PSII) is one of the major transmembrane protein supercomplexes in the thylakoid membrane of oxygenic photosynthesis, which initiates the light-driven electron transport chain reaction (Shevela et al. 2023). The core of the system consists of more than 20 proteins (Umena et al. 2011; Wei et al. 2016) which hold all major cofactors that participate in reactions that lead to the water splitting in PSII.

Upon the transfer of light energy from the antenna pigments to the PSII core, a charge separation takes place between P680, the primary electron donor and pheophytin, the primary electron acceptor. The charge separation is then stabilized by further electron transfer to Q_A_ and to Q_B_, the first and secondary quinone acceptors. After two consecutive charge separations, Q_B_ is protonated and released from its site to be replaced by the oxidized plastoquinole (Cardona et al. 2012; Müh et al. 2012; Kern et al. 2018). On the donor side of PSII, P680^+^ is reduced by the redox active tyrosine 161 on the D1 protein (Tyr_Z_) which, in turn, is reduced by the electron transfer from the CaMn_4_O_5_-cluster, the site where water oxidation occurs (Dau and Zaharieva 2009; Vinyard and Brudvig 2017; Shevela et al. 2023). The time scale of these reactions ranges from a few psec for charge separation to a few msec for O_2_ release and Q_B_ binding (Dau and Zaharieva 2009; Cardona et al. 2012; Shevela et al. 2023).

The CaMn_4_O_5_-cluster together with Tyr_Z_, H_2_O, H^+^ and O_2_ channels constitute the water oxidizing complex (WOC) in PSII. The WOC must cycle through the five intermediate states denoted S_n_ (n = 0–4) driven by Tyr_Z_^•^ with positive charges stored in the CaMn_4_O_5_-cluster. S_0_ is the most reduced state, S_2_ and S_3_ are metastable states, S_4_ is a transient state where O_2_ formation and release takes place, and S_1_ is the state which is most stable in the dark (Dau and Zaharieva 2009; Renger 2012; Vinyard and Brudvig 2017; Shevela et al. 2023). The mechanism of water oxidation has been studied to great detail by using a number of spectroscopic and computational methods and a complete understanding of O_2_ formation is near (Bhowmick et al. 2023; Greife et al. 2023). However, many issues still remain debated, including, for example, the molecular events during the S_2_ to the S_3_ state transition and the nature of the O=O bond formation (Suga et al. 2017; Kern et al. 2018; Bhowmick et al. 2023).

**Fig. 1:**
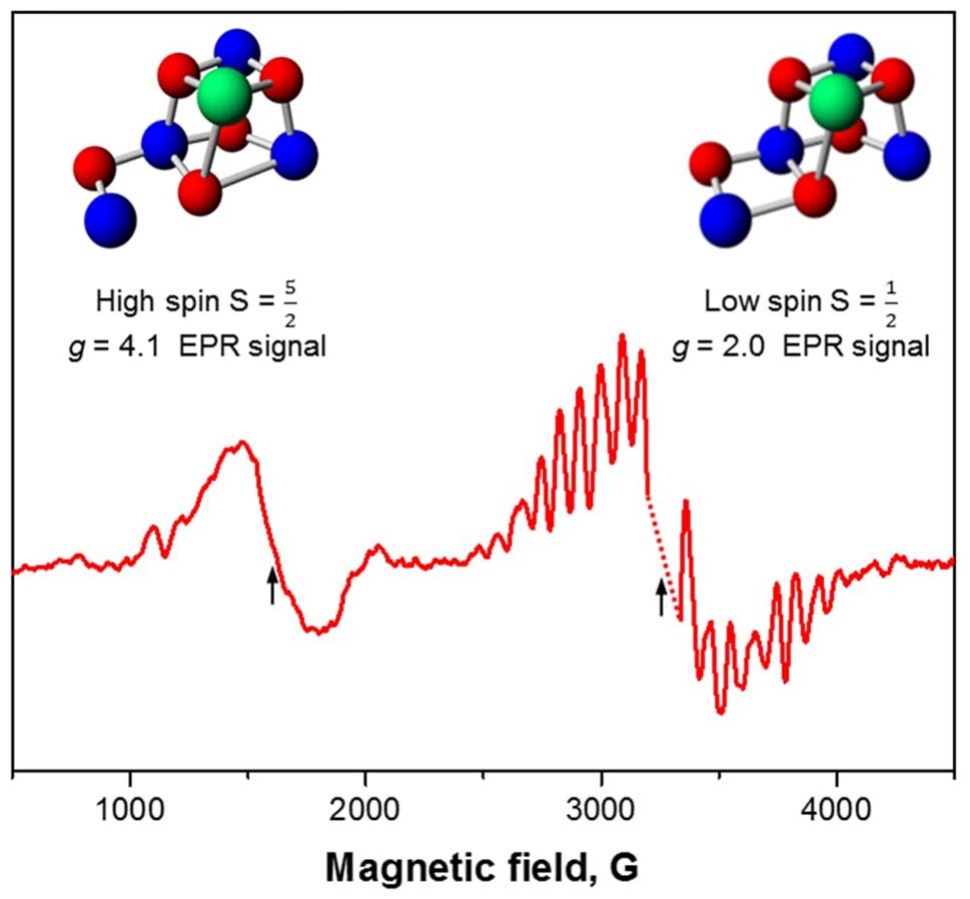
The low and high spin EPR signals from the S_2_ state of the WOC in PSII and corresponding computed structures of the CaMn_4_O_5_-cluster (Pantazis et al. 2012; Bovi et al. 2013)

The S_1_ → S_2_ state transition is the simplest one, and it has been long established that this transition only involves an electron transfer event (Rappaport and Lavergne 1991; Bernat et al. 2002; Boussac et al. 2022). By contrast, in the subsequent S_2_ → S_3_ transition a proton is expelled into the lumen and the binding of one water molecule takes place and leads to the formation of a sixth oxo bridge (Ugur et al. 2016; Suga et al. 2017; Wang et al. 2017; Kern et al. 2018; Siegbahn 2018; Mäusle et al. 2020; Navarro et al. 2013; Ibrahim et al. 2020; Takemoto et al. 2019; Kim and Debus 2019; de Lichtenberg et al. 2021). The deprotonation event is believed to precede the electron transfer, see Pantazis (2018) for references and extensive discussion. In plant PSII, the starting S_2_ state exhibits two distinct electron paramagnetic resonance (EPR) signals: the low spin $\frac{1}{2},$ g = 2 multiline signal (Dismukes and Siderer 1981) and high spin $\frac{5}{2},$ g = 4.1 signal (Zimmermann and Rutherford 1984; Casey and Sauer 1984; Depaula et al. 1985) (Fig. 1) or more complex > $\frac{5}{2}$ high spin signals at higher g values (Boussac et al. 1998a, b; Boussac and Rutherford 2000; Pokhrel and Brudvig 2014).

From computational studies, it has been proposed that these two electronic configurations of the S_2_ state, the low and high spin states, correspond to the two, structurally different but energetically similar arrangements of the CaMn_4_O_5_-cluster. The low spin S_2_ state corresponds to the open cubane structure and the high spin S_2_ state was proposed to have a closed cubane structure (Pantazis et al. 2012; Bovi et al. 2013) (Fig. 1). It was also proposed that the S_2_ → S_3_ state transition occurs via the high spin intermediate (closed cubane) which is triggered by the formation of the Tyr_Z_^•^ radical (Narzi et al. 2014; Retegan et al. 2016). In addition, it is important to mention that the nature of the high spin state EPR signal from the S_2_ state is different in cyanobacterial PSII where g = 4.24 and 4.75 forms of these EPR signal were reported (Boussac et al. 2000; Boussac 2019) and that other structural proposals were made for the S_2_ high spin state, including a proton shift isomer (Corry and O'Malley 2020) and early water binding (de Lichtenberg and Messinger 2020). Extensive pH and temperature studies in cyanobacteria support that the high spin intermediate is a required intermediate during the S_2_ → S_3_ transition (S_2_^LS^ → [S_2_^HS^] → S_3_) (Boussac et al. 2018; Boussac 2019), while it was not detected by XFEL studies (Kern et al. 2018; Bhowmick et al. 2023).

In this study we apply EPR spectroscopy to study the S_1_ → S_2_ and S_2_ → S_3_ state transitions in plant PSII. For investigating the S_2_ → S_3_ transition, the S_2_ state was poised in both the low spin and high spin EPR configurations. We discuss our data with respect to and in mechanistic comparison with results obtained from cyanobacterial preparations.

## Methods

### PSII membranes and EPR samples preparation

Spinach (*Spinacia oleracia*) was grown hydroponically as described previously at 20 °C under cool white fluorescent light (Osram Powerstar HQI-400W/DV dysprosium lamp, intensity 300 μE/m^2^/s), with light–dark periods of 12 h (Danielsson et al. 2004). Oxygen evolving PSII enriched membranes (BBY-type) were prepared according to previously published procedures (Völker et al. 1985). The membrane particles were re-suspended in a buffer containing 400 mM sucrose, 15 mM NaCl, 3 mM MgCl_2_ and 25 mM MES–NaOH pH 6.1, frozen as beads and stored at − 80 °C, at a chlorophyll (Chl) concentration of 6 mg/ml before use. When indicated, this sucrose buffer was used during the measurements.

For EPR samples preparation, PSII membranes were thawed, washed and diluted to a final concentration of 2 mg Chl/ml. The washing and dilution were made either in the sucrose buffer or in the buffer without sucrose but supplemented with 50% of ethylene glycol. When indicated, 3.5% methanol was added (see details in the corresponding figure legends). The diluted PSII membranes were then transferred to calibrated EPR tubes and pre-flashed according to (Han et al. 2008, 2012, 2022) with addition of the electron acceptor PpBQ dissolved in DMSO (1 mM final concentration) in order to achieve full synchronization of samples in the S_1_ state. Then samples were immediately frozen first in the ethanol/dry ice bath at 200 K and then transferred to liquid nitrogen at 77 K and stored overnight.

### Temperature dependence of the S_1_ → S_2_ and S_2_ → S_3_ state transitions

**Fig. 2:**
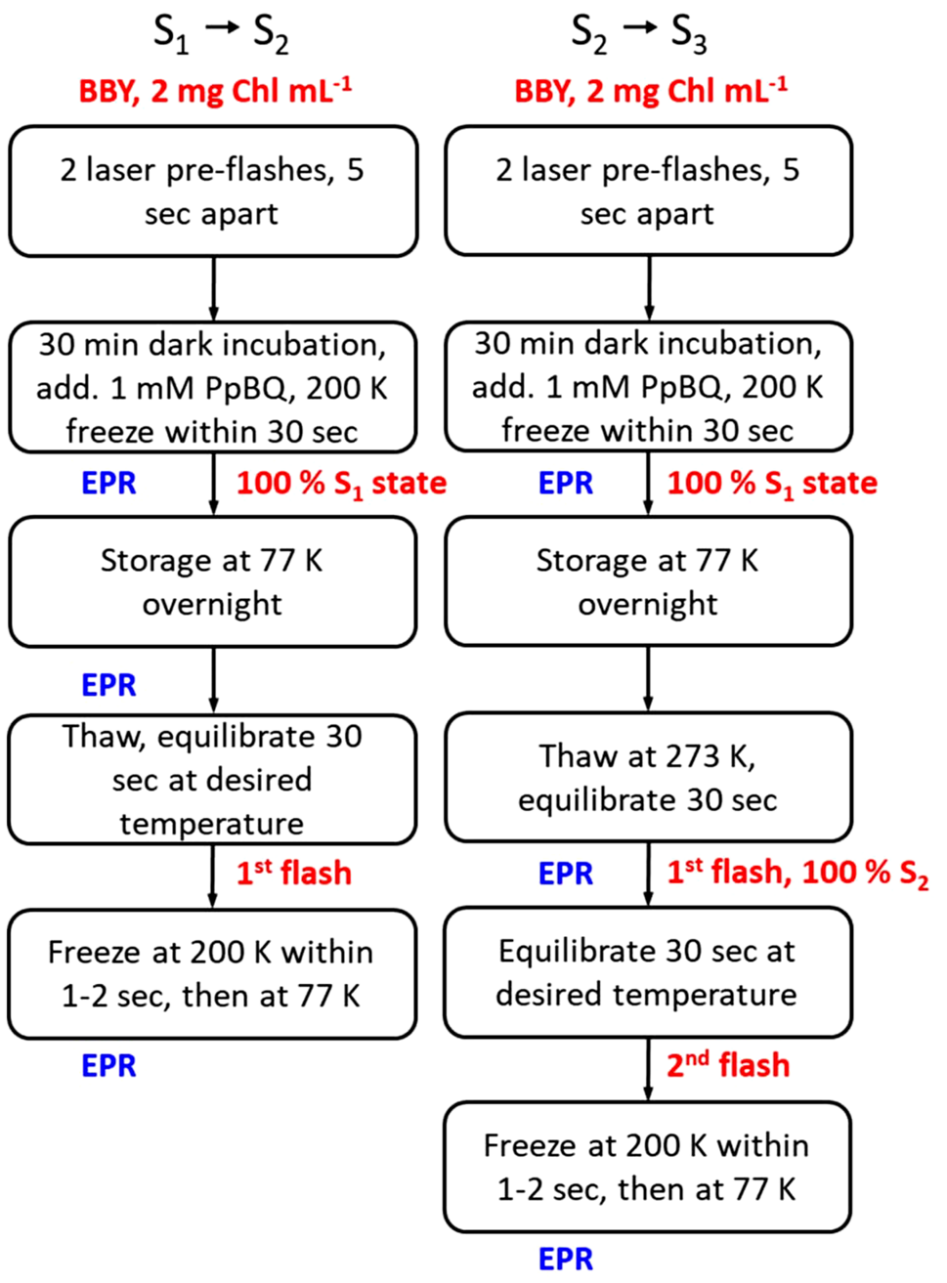
The pre-flash procedure, turnover flashes and temperature conditions employed in this study

To study the S_1_ → S_2_ state transition at different temperatures, on the next day, frozen EPR tubes with PSII sample in the S_1_ state were quickly thawed and incubated for 30 s at the desired temperature and given one saturating laser flash to advance to the S_2_ state. Immediately after the flash, samples were frozen first within 1–2 s at 200 K and then transferred to 77 K. After that EPR spectra were measured (Fig. 2).

To study the S_2_ → S_3_ state transition at different temperatures, frozen EPR tubes with PSII sample in the S_1_ state were quickly thawed to 273 K and given one laser flash to advance to the S_2_ state (100% of the S_1_ → S_2_ state transition, Han et al. 2008, Han et al. 2012, Han et al. 2022). Immediately after the first flash, samples were incubated for 30 s at the desired temperature. The second saturating laser flash was given at this temperature to advance PSII samples to the S_3_ state and were immediately frozen first at 200 K and then transferred to 77 K and EPR spectra were measured (Fig. 2).

In addition, to study the S_2_ → S_3_ state transition at different temperatures, the Split S3 EPR signal was induced by continuous near-infrared illumination for 30 min (830 nm LQC830-135 diode laser, Newport, USA, 160 W/m^2^) directly into the EPR cavity cooled to 5 K as described in (Han et al. 2008, 2012, 2022; Havelius et al. 2011). The complete flashing and temperature procedures are described in Fig. 2.

77 K temperature was achieved in the liquid N_2_ bath. Temperatures between 130 and 170 K were achieved in an isopentane bath and between 165 and 290 K in an ethanol bath (Styring and Rutherford 1988). Saturating laser flashes of 6 ns duration at 532 nm and 840 mJ power were provided by a Spectra Physics PRO-290 Q-switched Nd:YAG laser.

### EPR spectroscopy

EPR spectra were recorded with an ELEXSYS E500 spectrometer equipped with a SuperX bridge and a SHQ-4122 cavity (Bruker Biospin GmbH). All measurements were performed at liquid helium temperatures which were achieved with an ESR-900 cryostat and ITC-503 temperature controller, Oxford Instrument Ltd. Spectrometer settings for each spectrum are indicated in the figure legends. The standard error in our EPR measurements was less than 5%. Analysis of the EPR spectra was carried out with the Bruker Xepr 2.4b software.

## Results

### Temperature dependence of the S_1_ → S_2_ state transition

**Fig. 3:**
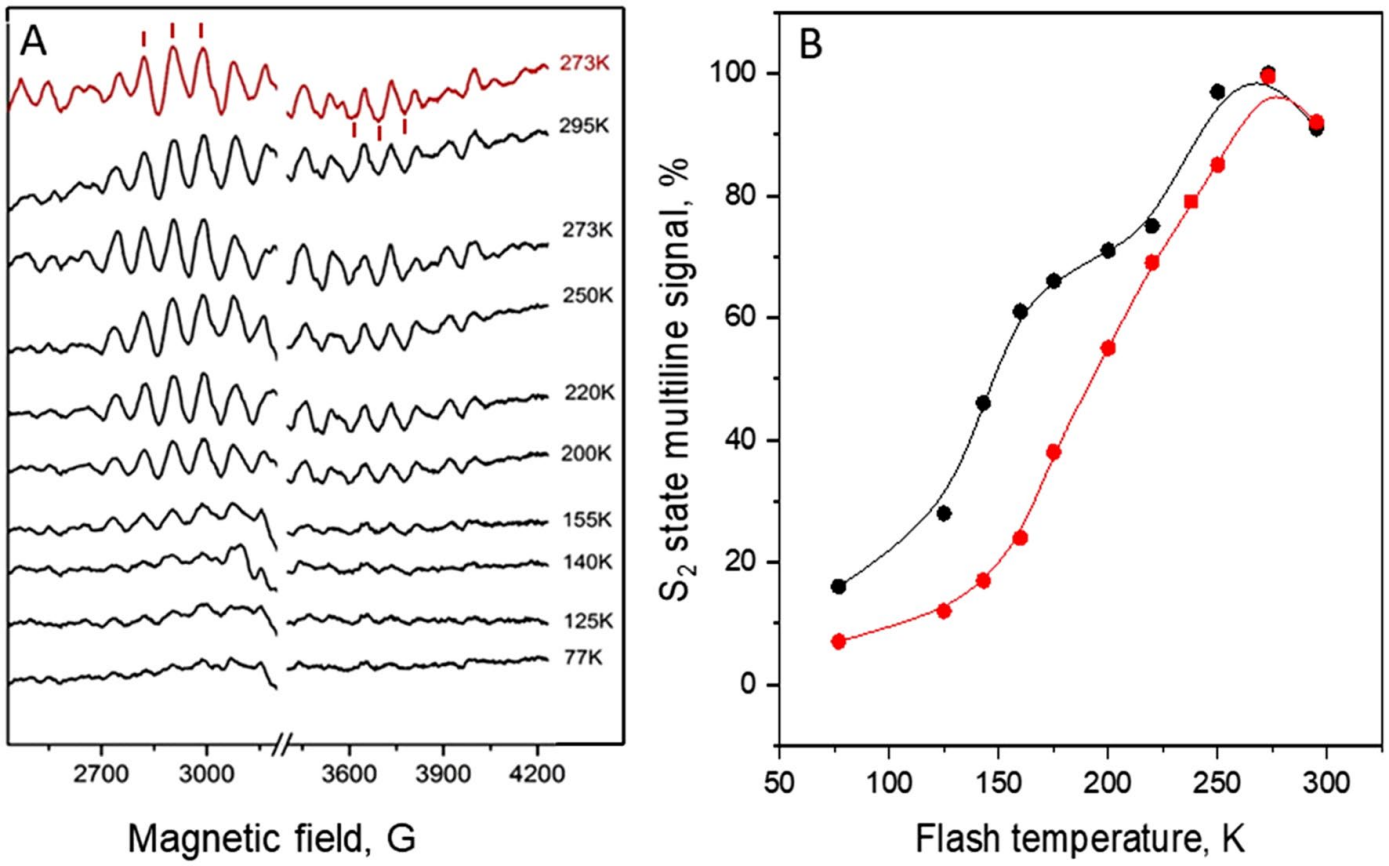
**A** One flash minus dark S_2_ state multiline EPR difference spectra in a sucrose buffer in the presence of 3.5% methanol. The red spectrum was obtained after flash was given at 273 K and was additionally illuminated at 200 K for 4 min. The large intensity from Tyr_D_^•^ at g = 2.0 is removed for clarity. The red bars indicate peaks used for the signal quantification. EPR conditions: microwave frequency 9.46 GHz, microwave power 10 mW, modulation amplitude 20 G, temperature 7 K. **B** Temperature dependence of the S_2_ state multiline signal formation in a 50% ethylene glycol buffer (black symbols) and in a sucrose buffer (red symbols) both containing 3.5% methanol (see Table 1). Data were normalized to the signal obtained at 273 K where 100% of transition occurs

In the presence of a few percent methanol, the only S_2_ state EPR signal from the CaMn_4_O_5_-cluster which is observable is the S = $\frac{1}{2}$ multiline signal (Deak et al. 1999). Formation of this signal in a sucrose buffer at sample temperatures in the range of 77–295 K in the presence of 3.5% methanol and 1 mM PpBQ is shown in Fig. 3A and its temperature dependence is shown in Fig. 3B (red symbols). After application of one saturating laser flash to the S_1_ state synchronized EPR samples at 273 K, a complete transition to the S_2_ state took place resulting in 100% of the S_2_ state (Han et al. 2008, 2012, 2022) as also could be seen from the size of red spectrum. At 295 K the transition was less than 100% due to the faster recombination reaction at this temperature (Han et al. 2008, 2012, 2022). Upon lowering the temperature below 273 K, the transition was less and less efficient (Fig. 3A). The half-inhibition temperature (T_0.5_) for the S_2_ state multiline EPR signal formation was found to be 185 K (Fig. 3B; red symbols), which is about 30 K higher than in our recent report where the same measurements were done in ethylene glycol buffer containing methanol (Pavlou et al. 2023) and even 50 K higher than in the original study by Styring and Rutherford (1988) performed in ethylene glycol buffer in absence of methanol (see Table 1). These data suggest that while methanol increases T_0.5_, ethylene glycol appears to strongly decrease T_0.5_ as compared to samples in sucrose buffer, which is usually assumed to be benign. In order to confirm the ethylene glycol effect, we repeated our earlier experiments (Pavlou et al. 2023) with 50% of ethylene glycol and 3.5% of methanol. The results are shown in Fig. 3B, black symbols. The T_0.5_ for the S_1_ → S_2_ transition was decreased significantly to 154 K, similar to what we reported before, Table 1. It is worth to mention that, when a single saturating laser flash was given at 77 K, we still observed 10–20% of the multiline signal formation at this temperature (Fig. 3A).

**Table 1:** Half inhibition T_0.5_ and full inhibition T_inh_ temperatures for the S_1_ → S_2_ state transition in PSII membranes in the different buffer composition

Study	T_0.5_ (K)	T_inh_ (K)	Buffer composition
Styring and Rutherford (1988)	135–140	< 77	Ethylene glycol, 50%
Pavlou et al. (2023)	157	< 77	Ethylene glycol, 50%
			Methanol, 3.5%
This study	154	< 77	Ethylene glycol, 50%
			Methanol, 3.5 %
This study	185	< 77	Sucrose, 400 mM
			Methanol, 3.5%

Interestingly, the temperature dependence in the presence of ethylene glycol exhibits a plateau at 160–220 K (Fig. 3B, see also Fig. 2 in Pavlou et al. 2023), above which the temperature dependence becomes independent of the type of cryoprotectant present (Fig. 3B). This was not observed in the original study (Styring and Rutherford 1988) since the highest transition temperature point measured was 200 K. The presence of this plateau, may indicate that at this temperature range a membrane phase transition occurs in presence of ethylene glycol in the buffer, which influences the PSII complex and resulting in a change of the temperature dependence of the S_2_ state multiline EPR signal formation.

### Temperature dependence of the S_2_ → S_3_ state transition in the presence of methanol

**Fig. 4:**
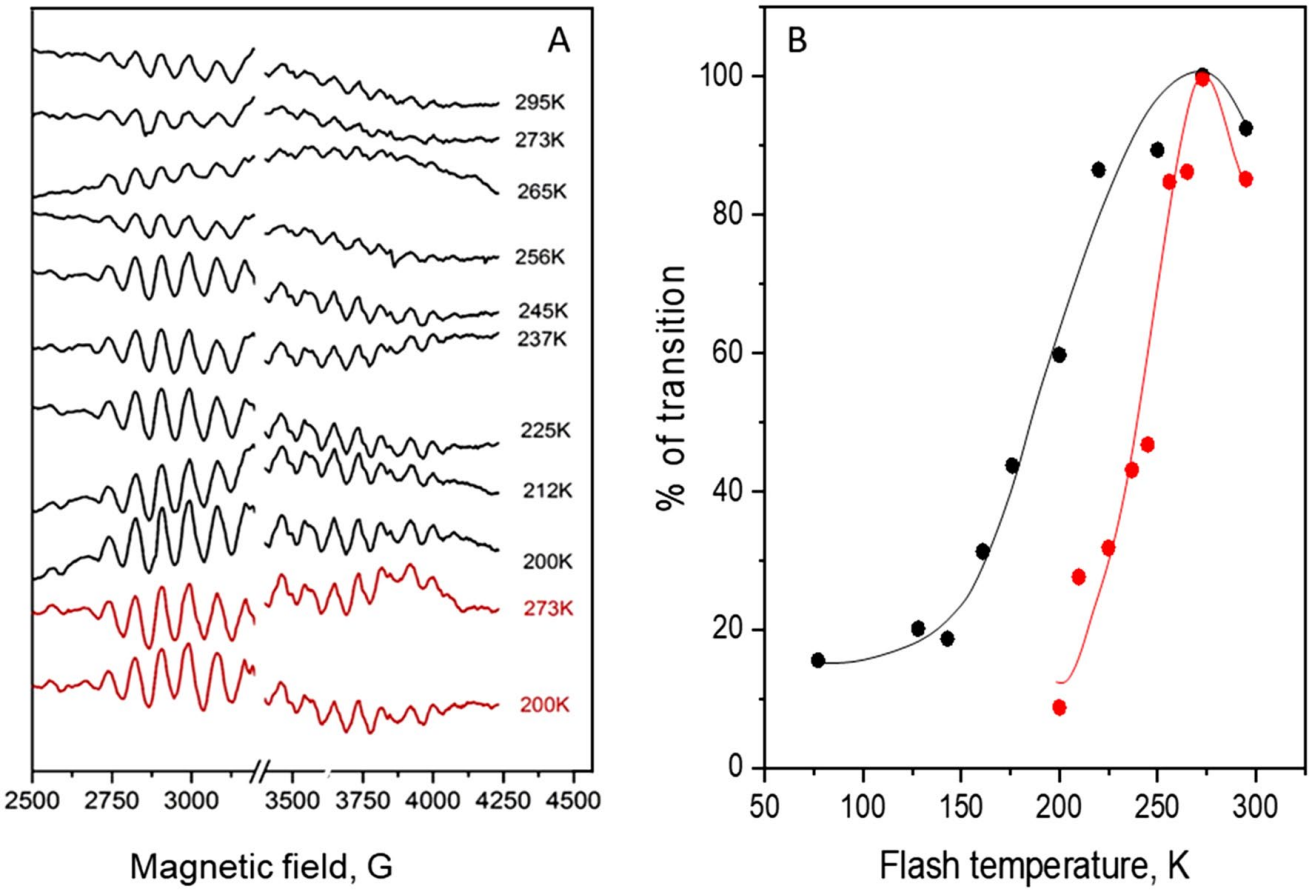
**A** Two flashes minus dark multiline difference EPR spectra showing the remaining S_2_ state in a sucrose buffer in the presence of 3.5% methanol after second flash was given at different temperatures. The red spectra represent the spectrum obtained after 1 flash at 273 K and followed by additional illumination at 200 K, i.e. the maximal S_2_ state signal. The large intensity from Tyr_D_^•^ at g = 2.0 is removed for clarity. EPR conditions: microwave frequency 9.46 GHz, microwave power 10 mW, modulation amplitude 20 G, temperature 7 K. **B** Temperature dependence of the S_1_ → S_2_ state transition (black symbols) and S_2_ → S_3_ state transition (red symbols) in a sucrose buffer in the presence of 3.5% methanol. Data were normalized to 100% at 273 K where the maximal transition occurs

In our next step we investigated the temperature dependence of the S_2_ to S_3_ state transition in the sucrose buffer in the presence of 3.5% methanol (see Fig. 2 for the procedure). The first laser flash was given at 273 K, allowing formation of the S_2_ state in 100% of the PSII centers (Han et al. 2008, 2012, 2022). Formation of the S_3_ state was estimated from the disappearance of the S_2_ state multiline EPR signal (Fig. 4A) after the second flash was given at the desired temperature. The maximal transition was again observed when second flash was given at 273 K (Fig. 4A, B) where more than 70% of the PSII centers turned over to the S_3_ state. As expected, the transition was less efficient at lower temperatures displaying a T_0.5_ of about 240 K, which is only 10 K higher than reported for the ethylene glycol containing samples without methanol (230 K, Styring and Rutherford 1988). For comparison, the temperature dependence for the S_1_ → S_2_ transition, obtained under the same conditions (i.e. in the sucrose buffer with 3.5% methanol) is also shown in Fig. 4B, black symbols. It is clear that difference in T_0.5_ between the S_1_ → S_2_ and S_2_ → S_3_ transitions in the presence of only methanol is 55 K, much less than was reported for PSII membrane samples containing 50% ethylene glycol (90–95 K) (Styring and Rutherford 1988).

### Temperature dependence of the S_2_ → S_3_ state transition in the absence of methanol

**Fig. 5:**
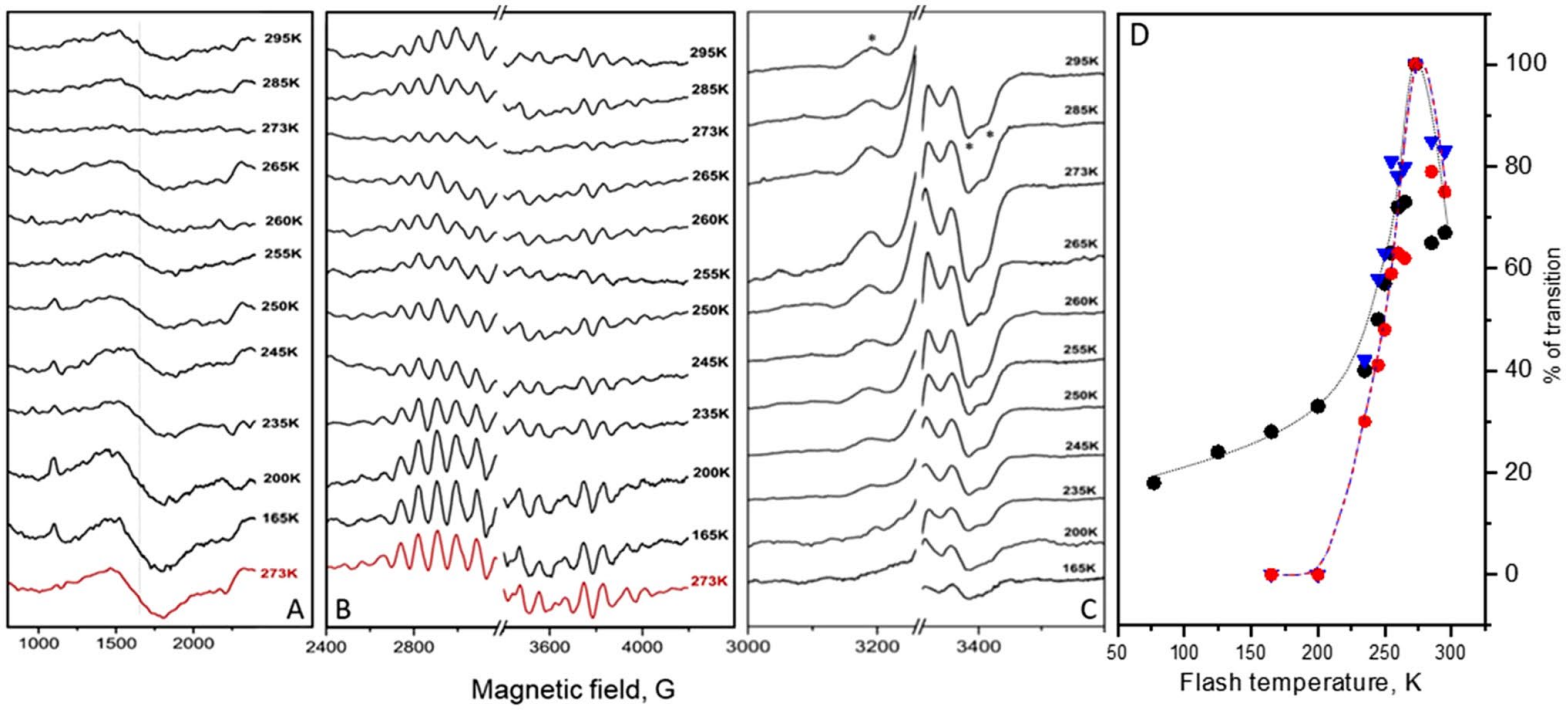
**A** Two flashes minus dark difference spectra showing remaining S_2_ state g = 4.1 and **B** multiline EPR signals in a sucrose buffer in the absence of methanol after the second flash was given at the indicated temperatures. The red spectra were obtained after one flash at 273 K, i.e. they show the maximal S_2_ state signal. **C** Light minus dark difference spectra of the Split S_3_ EPR signal induced after the second flash was given at the indicated temperatures. The signal was induced by NIR illumination at 830 nm for 30 min at 5 K. The large intensity in B and C from Tyr_D_^•^ at g = 2.0 is removed for clarity. The asterisks indicate peak and troughs used for the signal quantification. **D** Temperature dependence of the S_2_ → S_3_ transition in a sucrose buffer in the absence of methanol, based on spectra shown in **A**–**C**, the S_2_ multiline EPR signal (red symbols), g = 4.1 EPR signal (blue symbols) and the Split S_3_ EPR signal (black symbols). Data were normalized to 100% at 273 K where the maximal transition occurs. EPR conditions: microwave frequency 9.46 GHz, microwave power 32 mW in **A** and **B** and 25 mW in **C**; modulation amplitude 20 G in **A** and **B**; and 15 G in **C**, temperature 9 K in **A** and **B** and 5 K in **C**

EPR signals originated from the WOC are known to be very sensitive to the small structural alterations which could be induced by differences in the buffer composition (Boussac and Rutherford 2000; Pokhrel and Brudvig 2014). Addition of few percent of methanol is known to produce such effects; it enhances the low spin g = 2 multiline EPR signal and fully suppresses the high spin g = 4.1 EPR signal (Deak et al. 1999). It is also known to completely eliminate or modify the so-called Split S state EPR signals (Su et al. 2006). In order to investigate the S_2_ → S_3_ state transition where the starting S_2_ state contains both low and high spin electronic configurations of the CaMn_4_O_5_-cluster, we performed our next experiments in a sucrose containing buffer with 0% methanol. At these conditions, after the first flash given at 273 K, the S_2_ state exhibited both the g = 2 multiline and g = 4.1 EPR signals (Figs. 1, 5A, B). Estimation on the basis of the amplitude of the methanol containing multiline signal shows that, after one flash, 52% of the PSII centers were in the low spin configuration (S_2_ state multiline) and 48% in the high spin configuration (S_2_ state g = 4.1).

After application of the second flash, the S_2_ → S_3_ transition took place and the efficiency of this transition with respect to the initial S_2_ state configuration was monitored by disappearance of the respective EPR signals. Figure 5A–B show the second flash minus dark difference spectra obtained at second flash temperatures in the range of 165–295 K. For the g = 4.1 signal, the maximum transition was achieved at 273 K where more than 75% of the signal disappeared (Fig. 5A). The signal amplitude, remaining after the second flash at this temperature, was only 23% of the maximal g = 4.1 EPR signal obtained after the first flash (Fig. 5A, red spectrum). At higher temperatures, the transition efficiency was lower and the remaining amplitude of the g = 4.1 signal was 36% and 40% after second flash was given at 285 and 295 K, respectively (Fig. 5A). Upon decreasing the temperature of the S_2_ → S_3_ transition from 273 K, the amplitude of the remaining g = 4.1 signal after the flash is gradually increased, indicating less and less efficient transition. The remaining g = 4.1 signal was found to be 77% at 235 K and reached 100% at 200 K (Fig. 5A). Thus, at 200 K and below the S_2_ → S_3_ transition was effectively blocked.

The temperature dependence of the S_2_ → S_3_ transition was also monitored by tracking the disappearance of the S_2_ state g = 2 multiline EPR signal (Fig. 5B). The results were essentially the same as for the g = 4.1 signal, as indicated by the comparison of their temperature dependences in Fig. 5D and similar to what was reported before in presence of ethylene glycol and absence of methanol (Styring and Rutherford 1988).

In order to further investigate the S_2_ → S_3_ transition at different temperatures, we also measured the formation of the S_3_ state. The S_3_ state of the WOC is characterized by several EPR signals (Boussac et al. 2009; Havelius et al. 2010; Petrouleas et al. 2005; Matsukawa et al. 1999; Ioannidis and Petrouleas 2000; Ioannidis et al. 2002). We used the so-called Split S_3_ signal, obtained by the infrared light illumination of the S_3_ state at 5 K. The signal originates from the magnetic interaction of the CaMn_4_O_5_-cluster in the S_3_ state and Tyr_Z_^•^ radical at cryogenic temperatures (Petrouleas et al. 2005; Havelius et al. 2010, 2011) and reports quantitatively on the amount of the S_3_ state (Han et al. 2008). The signal is characterized by the broad peak at g = 2.03 (~ 3200 G) and the double trough at g = 2.01 and 1.99 (~ 3390 and ~ 3410 G), Fig. 5C.

Above 230 K, the Split S_3_ signal formation followed very closely the temperature dependence of the disappearance of the S_2_^HS^ and S_2_^LS^ signals (Fig. 5C, D). Interestingly, below 230 K, the traces deviate (Fig. 5D) because the Split S_3_ signal was still observable at the level of 20–30% down to 77 K, while on the basis of both S_2_ signals the S_2_ → S_3_ transition appears to be fully blocked. Normalized temperature dependences of the S_2_ → S_3_ transition from all measurements are shown in Fig. 5C. It is clear that in all three cases the T_0.5_ was found to be about 240 K.

## Discussion

Our data show that the inhibition temperature of the S_1_ → S_2_ state transition is strongly influenced by the buffer composition and the presence of methanol (Table 1). The lowering of its half-inhibition temperature by ethylene glycol is difficult to assign to the specific site and most probably is a general effect, possibly reflecting the greater ability of ethylene glycol compared to sucrose to keep protein motions and water mobility active down to lower temperatures. In contrast, methanol is a small molecule, which is known to interact with the CaMn_4_O_5_-cluster and change its spectroscopic properties (Messinger et al. 1997; Åhrling et al. 1997, 2006; Force et al. 1998; Deak et al. 1999; Su et al. 2006; Oyala et al. 2014; Retegan and Pantazis 2016; Nagashima and Mino 2017; Yata and Noguchi 2018; Zahariou et al. 2021; Kalendra et al. 2022). It has been suggested to bind close to either Mn_1_ or Mn_4_, thus disturbing the H-bonding network around the cluster (Retegan and Pantazis 2016; Nagashima and Mino 2017; Kalendra et al. 2022). Considering that the “dangling” Mn_4_ is oxidized during the S_1_ to S_2_ state transition (Mn^3+^ to Mn^4+^) (Kern et al. 2018), the possibility of methanol binding and disturbance of the water network around Mn_4_ is more plausible and could certainly shift the transition temperature of this oxidation step to a higher temperature. It is noted that while in the S_1_ → S_2_ transition no proton is released from PSII, an internal proton movement from for example water 2 to a protein site or oxo bridge have been proposed (Shoji et al. 2019; Corry and O'Malley 2019). The strong sensitivity of T_0.5_ on methanol is in line with these ideas.

Another interesting observation for the S_1_ → S_2_ transition, based on the multiline signal measurements is the formation of 10–15% of the signal at very low temperatures down to 77 K (Table 1; Figs. 3B, 4B) (Pavlou et al. 2023). The nature of this small fraction of PSII centers, capable of such low temperature electron transfer is unclear and requires further investigation. One speculation could be that it is connected to an S_1_ state heterogeneity, such as a flip of the Jan Teller axis of the Mn_4_ ion (Drosou et al. 2021) or the presence of a protonation isomer of the CaMn_4_O_5_-cluster. In the letter scenario it seems possible that PSII centers in which water 2 is deprotonated can transition to S_2_ even at 77 K, while those in which water 2 is protonated require conditions under which such transfer to a yet unknown internal base is possible.

The S_2_ → S_3_ transition is more complicated with a deprotonation event, substrate water binding, and insertion of Ox into the cluster (Ugur et al. 2016; Wang et al. 2017; Kern et al. 2018; Siegbahn 2018; Ibrahim et al. 2020; Hussein et al. 2021). Formation of Tyr_Z_^•^ initiates this reaction by prompting deprotonation, which precedes the electron transfer (see Pantazis 2018 and references therein). Surprisingly, our data and comparison to literature show that there is practically no effect of ethylene glycol or methanol addition on T_0.5_ of this transition (Figs. 4, 5). The different sensitivity of the two transitions to ethylene glycol and methanol is in qualitative agreement with methanol binding near Mn_4_, since water binding occurs at Mn_1_ and it may be more difficult for MeOH to alter this transition from the distance (Umena et al. 2011; Suga et al. 2017; Kern et al. 2018; Ibrahim et al. 2020; Hussein et al. 2021).

In the absence of methanol two different S_2_ state EPR signals are observed, the low spin g = 2 multiline and high spin g = 4.1 signal (Fig. 1) which allowed us to study the S_2_ → S_3_ state transition relying on two signals with different electronic configuration (Zimmermann and Rutherford 1984; Casey and Sauer 1984; Depaula et al. 1985). We studied the disappearance of these signals after the second flash was given at different temperatures (Fig. 5A, B). Both low and high spin signals disappeared with the same temperature dependence with T_0.5_ at 240 K (Fig. 5D). We assume that if there are any EPR visible intermediates during the transition to the S_3_ state, it would be possible to detect these intermediates at lower temperatures. However, we were unable to observe any such intermediates. Thus, our results favor the conclusion that, in PSII isolated from spinach, both low spin g = 2.0 and high spin g = 4.1 configurations independently proceed to the S_3_ state in a similar fashion.

An interesting result from the Split S_3_ signal measurements is that this signal was still inducible in samples where the second flash was given at temperatures below 200 K and no disappearance of the S_2_ state related signals was observed (Fig. 5). The fraction of PSII centers which showed this phenomenon was ca. 20% (Fig. 5D). To understand how this is possible we need to consider the nature of the Split S_3_ EPR signal. In contrast to split signals from other S states, which could be generated only by the visible light illumination at 5 K and require Tyr_Z_ oxidation by charge separation in PSII (Petrouleas et al. 2005; Havelius et al. 2010), the Split S_3_ signal is generated also by NIR light and is believed not to involve the primary charge separation (Havelius et al. 2011; Boussac et al. 2008; Koulougliotis et al. 2003). It was suggested that the near-infrared excitation of one of the Mn^4+^ ions in the CaMn_4_O_5_-cluster leads to a reverse electron transfer from Tyr_Z_ to the CaMn_4_O_5_-cluster in the S_3_ state and, thereby, to the formation of the modified ${\text{S}}_{{2}}^{\prime }$ Tyr_Z_^•^ state that gives rise to the Split S_3_ EPR signal (Havelius et al. 2011). Therefore, it is very likely that this ca. 20% fraction of PSII centers that we observe as a Split S_3_ signal without actually advancing to the S_3_ state reflects an H^+^ equilibrium at the site and originates from a deprotonated form of the S_2_ state, ${\text{S}}_{{2}}^{\prime }$.

It is important to compare and discuss our findings regarding the S_2_ → S_3_ transition with respect to the published data from both spinach (Chrysina et al. 2010) and cyanobacterial PSII preparations from *T. elongatus* (Boussac et al. 2018; Boussac 2019). Using a similar spinach preparation and probing the S_2_ → S_3_ transition at 223 K and 243 K, Chrysina et al. (2010) reached the conclusion that the high spin g = 4.1 signal first converts to the low spin multiline signal which, in turn, proceeds to the S_3_ state. This conclusion was based on the accumulation of additional multiline signal at the expense of the g = 4.1 signal during a train of 1–2 ms xenon lamp flashes. In contrast, we used here saturating laser flashes and after the first flash given at 273 K all PSII centers have advanced to the S_2_ state (Fig. 5A, B) (Han et al. 2008, 2012, 2022). Additionally, we found that both spin states advance with similar efficiency in the range of 165–295 K. Further studies are required to understand this discrepancy.

Employing PSII core preparations from *T. elongatus* (now referred to *T. vestitus*) Boussac and coworkers concluded that the high spin S_2_ state is a deprotonated form of the low spin S_2_ state (working down to 230 K) and is most likely an intermediate on the way to the S_3_ state. They found that the deprotonated form can advance to S_3_ at temperatures down to 198 K (Boussac et al. 2018; Boussac 2019). This is different to what we observe in our plant PSII preparations where both forms of the S_2_ state, including the high spin form, don’t “move” forward at this temperature (Fig. 5). The reason for this discrepancy is unclear at the moment, but is most likely related to important differences in the S_2_^HS^ states. The high spin S_2_ state from cyanobacterial PSII exhibits an EPR signal with a shifted g value at 4.75, if compared with plant signal at g = 4.1. The only report of a similar g value in plant was obtained with a spinach PSII preparation in which the three extrinsic subunits had been removed (PsbO, PsbP and PsbQ) and a high concentration of Ca^2+^ was present in the buffer. For this sample, a high spin signal at g = 4.9 displaying a hyperfine structure was observed (Taguchi et al. 2020). The g = 4.9 signal was assigned to the S = $\frac{7}{2}$ excited state and to the structure with deprotonated water bound to Mn_4_ (Corry and O'Malley 2020). These differences could reflect slightly different H-bonding environment around the CaMn_4_O_5_-cluster between the cyanobacterial and plant PSII, making straightforward comparison of temperature dependencies between two species difficult.

In summary, our present data provide clear evidence that al low temperatures the formation of the S_2_ state from the S_1_ state is sensitive to both methanol and ethylene glycol addition, possibly indicating an internal proton transfer near Mn_4_. Furthermore, our data do not support the need of forming the high spin S_2_ state for advancing to the S_3_ state, which is in line with XFEL data (Kern et al. 18; Hussein et al. 2021).

## Data Availability

Not applicable.
